# The Ran Pathway in *Drosophila melanogaster* Mitosis

**DOI:** 10.3389/fcell.2015.00074

**Published:** 2015-11-26

**Authors:** Jack W. C. Chen, Amy R. Barker, James G. Wakefield

**Affiliations:** ^1^Biosciences, College of Life and Environmental Sciences, University of ExeterExeter, UK; ^2^Centre for Microvascular Research, William Harvey Research Institute, Barts and The London School of Medicine and Dentistry, Queen Mary University of LondonLondon, UK

**Keywords:** mitosis, RanGTP, microtubules, mitotic spindle, *Drosophila melanogaster*

## Abstract

Over the last two decades, the small GTPase Ran has emerged as a central regulator of both mitosis and meiosis, particularly in the generation, maintenance, and regulation of the microtubule (MT)-based bipolar spindle. Ran-regulated pathways in mitosis bear many similarities to the well-characterized functions of Ran in nuclear transport and, as with transport, the majority of these mitotic effects are mediated through affecting the physical interaction between karyopherins and Spindle Assembly Factors (SAFs)—a loose term describing proteins or protein complexes involved in spindle assembly through promoting nucleation, stabilization, and/or depolymerization of MTs, through anchoring MTs to specific structures such as centrosomes, chromatin or kinetochores, or through sliding MTs along each other to generate the force required to achieve bipolarity. As such, the Ran-mediated pathway represents a crucial functional module within the wider spindle assembly landscape. Research into mitosis using the model organism *Drosophila melanogaster* has contributed substantially to our understanding of centrosome and spindle function. However, in comparison to mammalian systems, very little is known about the contribution of Ran-mediated pathways in *Drosophila* mitosis. This article sets out to summarize our understanding of the roles of the Ran pathway components in *Drosophila* mitosis, focusing on the syncytial blastoderm embryo, arguing that it can provide important insights into the conserved functions on Ran during spindle formation.

## The core ran pathway in *Drosophila* embryos

### The *Drosophila* early embryo as a model system for mitotic spindle formation

**Figure 1:**
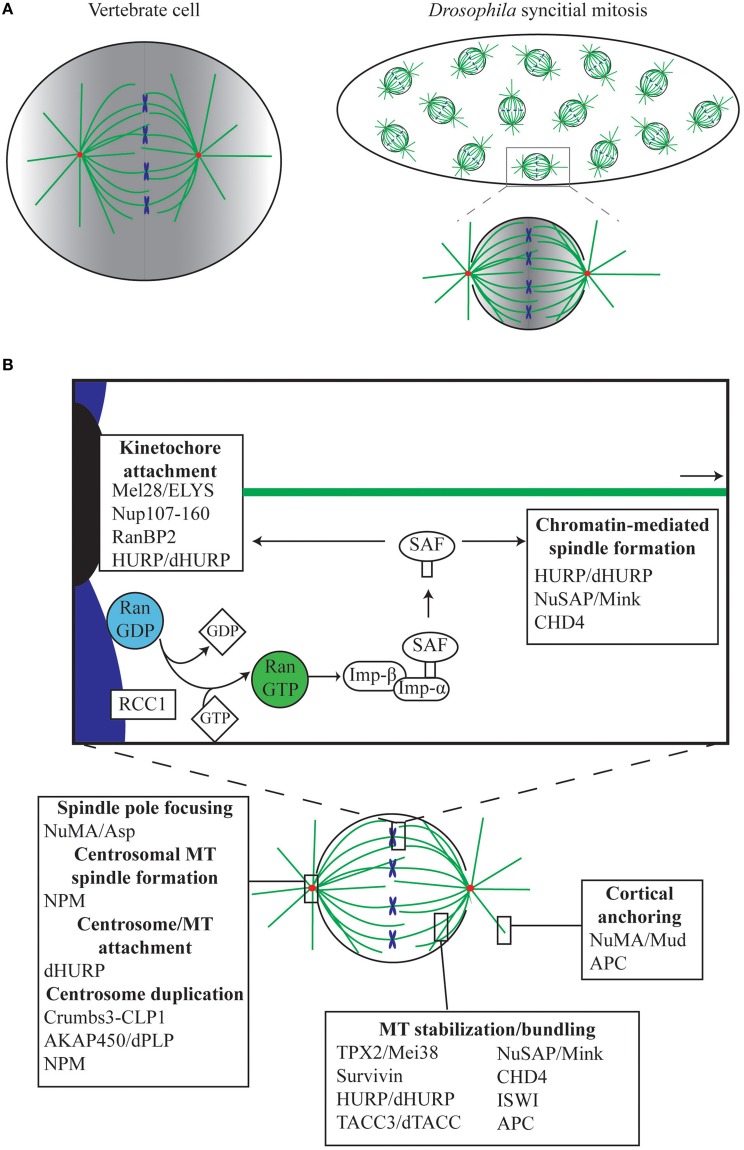
**The role of Ran in mitosis**. **(A)** The *Drosophila* syncytial embryo as a tool for understanding mitosis. In the *Drosophila* early embryo, the first 13 rounds of mitosis occur rapidly and take place in a shared cytoplasm. Unlike vertebrate cells, which undergo open mitosis and disassemble the nuclear envelope during mitosis, *Drosophila* undergoes semi-open mitosis, only disassembling the nuclear envelope at the spindle poles. Red, centrosomes; green, MTs; blue, chromosomes. In both vertebrates and *Drosophila*, Ran.GTP is generated in the vicinity of the chromatin, resulting in a gradient (shown in gray), which is strongest around the chromosomes and weakest at the poles and cortex. **(B)** Ran mediates mitotic functions via release of Spindle Assembly Factors (SAFs). During mitosis, Ran.GTP is generated around the chromosomes by the chromatin-bound RanGEF RCC1, facilitating the release of SAFs, which are otherwise sequestered by Importins (Imp-β/Imp-α). SAFs have critical roles in, amongst other things, MT anchoring to the kinetochores and centrosomes, in spindle growth from the chromatin, in MT bundling and stabilization, and in anchoring of astral MTs to the cell cortex.

With a fast generation time of 9 days at 25°C, relative ease of genetic manipulation, and a fully sequenced genome (Adams et al., 2000), *Drosophila* is a powerful model organism for studying basic biological processes such as mitosis. *Drosophila* tissues are an easily obtainable source material with which to investigate different types of cell division, including asymmetric cell division of neuroblasts, polarized mitosis of the ovarian epithelium, meiosis in the testes and oocytes, and syncytial mitosis in early embryos. In addition, *Drosophila* cell lines are available for *in vitro* cell culture techniques. Although *Drosophila* differs from vertebrate mitosis in that it undergoes semi-open mitosis (Figure 1A), where the nuclear envelope only fully breaks down at the spindle poles and where nuclear pores only fully dissociate at metaphase (Fuchs et al., 1983; Stafstrom and Staehelin, 1984), the overarching pathways involved in spindle formation are very similar.

Of the various tissues available, the *Drosophila* early embryo exhibits some additional advantages in teasing out fundamental concepts of spindle assembly. Following fertilization, the embryo undergoes 13 rounds of synchronous mitoses within a common cytoplasm (Figure 1A), before proceeding to cellularization. In contrast to the much longer time required for a full cell cycle in most animal cells, these syncytial cycles traverse through sequential S and M phases without intervening growth phases, with each round completed within 10–25 min (Foe and Alberts, 1983). During mitotic cycles 10–13 the nuclei are positioned close to the cortex of the embryo, and are therefore easy to image using confocal microscopy. Importantly, mitosis in the syncytium depends largely on maternally-supplied proteins, with the majority of transcription and translation silenced until cycle 14 (Foe and Alberts, 1983; Sullivan and Theurkauf, 1995; De Renzis et al., 2007; Lécuyer et al., 2007; Benoit et al., 2009). This, in conjunction with the shared cytoplasm and the large size of the embryo in relation to normal somatic cells (~150 μm long), means that the effects on spindle assembly of disrupting single proteins or protein complexes can be easily observed via microinjection-based methods (Brust-Mascher and Scholey, 2009; Conduit et al., 2015). Furthermore, embryonic mitoses appear to be much more tolerant to subtle perturbation than mammalian somatic cells, as syncytial nuclei continue to cycle, attempting to form spindles and segregate chromosomes, even in the presence of centrosome, spindle, or chromosome abnormalities (Hayward et al., 2014). However, although the syncytial embryo and *Drosophila* in general has contributed substantially to our understanding of canonical centrosome-driven spindle formation, very little is actually known in the fly about chromatin-driven spindle self-organization in which Ran plays such a central role. This review will outline the current understanding of the Ran pathway in *Drosophila*, and how it relates to the Ran pathway in other metazoans.

### Characteristics of ran during mitosis in non-*Drosophila* systems

**Figure 2:**
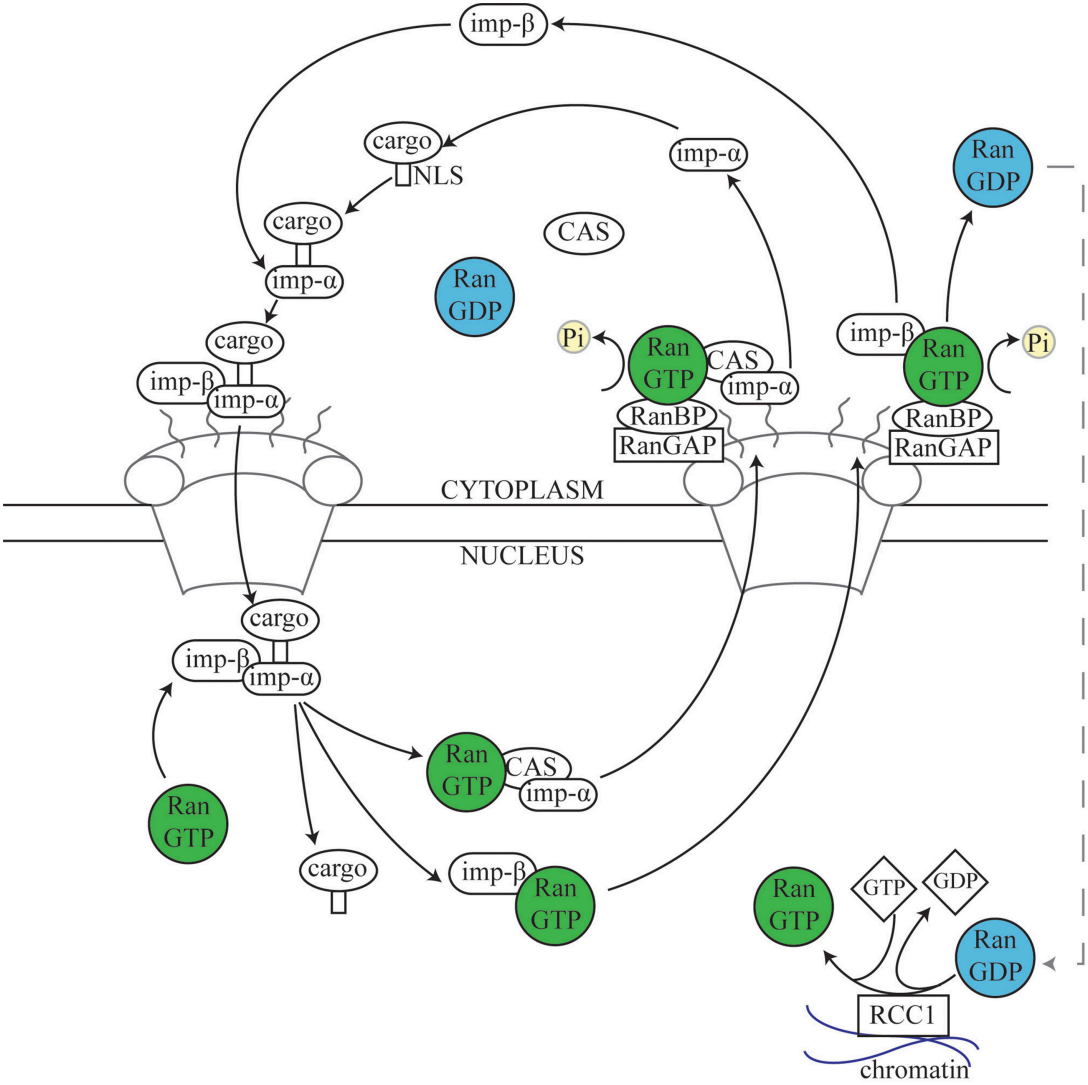
**The nuclear transport cycle**. During nuclear import, Importins in the cytoplasm recognize and bind to the Nuclear Localization Signal (NLS) of a target protein. The complex of cargo plus the Importin α/β dimer docks at the cytoplasmic side of the nuclear pore and is transported into the nucleus. Subsequently, Ran.GTP in the nucleus binds to Importin β, resulting in disassembly of the complex and releasing the cargo into the nuclear space. Importin α is then recycled back to the cytosol by the Exportin Cellular Apoptosis Susceptibility (Cas) protein via normal Ran-dependent export pathways (Kutay et al., 1997; Tekotte et al., 2002), while the Importin β/Ran.GTP complex is transported separately (Kose et al., 1999). Upon reaching the cytosolic side of the nuclear envelope, Ran.GTPase Activating Protein 1 (RanGAP1) and Ran Binding Proteins (RanBPs) stimulate Ran-dependent GTP hydrolysis, causing release of Importin β from Ran (Kutay et al., 1997; Lounsbury and Macara, 1997; Seewald et al., 2003). RanGAP1 is unable to directly affect Ran.GTP complexed with either importins or exportins, and instead acts via one of several Ran binding proteins (RanBPs) (Bischoff and Görlich, 1997; Floer et al., 1997; Kutay et al., 1997; Lounsbury and Macara, 1997). Nuclear export follows a similar process; Exportins such as chromosome region maintenance 1 (Crm1) recognize and bind to Nuclear Export Signals (NESs) on target proteins. Exportin, Ran.GTP, and cargo form a complex which passes out of the nucleus through the nuclear pore and, as described above (not shown) (Fornerod and Ohno, 2002; Kuersten et al., 2002). Cytosolic Ran.GDP is transported into the nucleus, where the chromatin-bound Ran guanine exchange factor (RanGEF) RCC1 re-generates a pool of Ran.GTP (Ohtsubo et al., 1989; Bischoff and Ponstingl, 1991; Klebe et al., 1995). Thus, the spatial restriction of RanGAP1, RanBPs, and RCC1 results in a large cytoplasmic pool of Ran.GDP and a large nuclear pool of Ran.GTP (Bischoff et al., 1994; Klebe et al., 1995).

Ran was first discovered as a substrate for the RCC1 Guanine nucleotide Exchange Factor (GEF; Bischoff and Ponstingl, 1991), and has been predominantly characterized as a central player in nuclear transport, shuttling proteins and mRNA into and out of the nucleus. It is a ~25 kDa guanosine triphosphatase (GTPase) related to the Ras superfamily of GTPases, and can exist in the active guanosine triphosphate-bound (GTP) state or the inactive guanosine diphosphate-bound (GDP) state. Although Ran has inherent GTPase activity, Ran binding proteins (RanBPs) and Ran GTPase activating protein (RanGAP) are essential for effective GTP hydrolysis to take place in a cellular context (Bischoff et al., 1994; Bischoff and Görlich, 1997). Nuclear transport has been well-characterized (Figure 2) and excellent reviews already exist (Stewart, 2007; Cautain et al., 2015). While these functions are outside the main scope of this review, it is important to consider the similarities between the role of Ran in nuclear transport and in mitosis.

The current model explaining how the Ran signal transduction pathway contributes to spindle assembly was originally conceptualized from observations in *Xenopus* embryo extracts (Carazo-Salas et al., 1999; Kalab et al., 1999; Ohba et al., 1999; Wilde and Zheng, 1999); indeed, the majority of studies to date on Ran function during mitosis have been carried out in *Xenopus* and verified in mammalian cell lines. Both systems utilize open mitosis, where the nuclear envelope breaks down fully and cytoplasmic and nuclear contents—including Ran—mingle during mitosis (Hutchins et al., 2009). RCC1 binds to chromatin (Ohtsubo et al., 1989; England et al., 2010), and through its interaction with Histones (Nemergut et al., 2001; Makde et al., 2010) and DNA (Chen et al., 2007), generates a locally high concentration of Ran.GTP (Bischoff and Ponstingl, 1991). As Ran.GTP diffuses away from the chromatin, RanGAP induces GTP hydrolysis, and Ran.GTP is converted to Ran.GDP (Kalab and Heald, 2008); an activity enhanced by RanBP1 (Seewald et al., 2003). As a result, a Ran.GTP gradient is formed throughout the cytoplasm with the highest concentration around the chromosomes. Ran.GTP acts to release a set of proteins termed Spindle Assembly Factors (SAFs) from their interaction with the Importin family of proteins [see Section The Role of Karyopherins (Importins and Exportins) in *Drosophila* Mitosis]. As these SAFs are locally released around the chromatin, it is in this region of the cell that they promote MT polymerization, stabilization and organization (Figure 1). The classic model of mitotic Ran function suggests that the Ran gradient has a particularly crucial role in generating MTs, and therefore spindles, independently of the classic MT organizing centers, centrosomes (Gruss et al., 2001; Wiese et al., 2001). For example, a bipolar spindle array can be generated from just chromatin-coated beads (Heald et al., 1996), or from beads coated with RCC1 in *Xenopus* embryo extract (Halpin et al., 2011), while activating Ran at the plasma membrane through specific targeting of a modified RCC1 results in ectopic MT aster generation (Zonis and Wilde, 2011). However, as described below, the localization of Ran to additional cellular locations during mitosis, and variations in the requirement of a Ran gradient in the generation of a mitotic spindle, suggests a more complex role for Ran than a simple gradient model suggests.

### A role for the ran pathway in the *Drosophila* syncytial embryo?

The clear dominance of centrosome-driven MT nucleation during *Drosophila* embryonic spindle formation, and differences in the presence or spatio-temporal localization of Ran pathway components between *Drosophila* and vertebrate mitosis, has left the role of Ran in *Drosophila* unclear. For example, in addition to localizing in a gradient around mitotic chromatin, Ran.GTP has also been described at both human mitotic centrosomes (Keryer et al., 2003), and mouse meiotic MTs (Cao et al., 2005), while in *Drosophila* embryos, Ran is found only throughout the spindle region; albeit in a diffuse gradient (Trieselmann and Wilde, 2002; Moutinho-Pereira et al., 2013). Similarly, the absence of a mitotic phenotype upon RNAi-mediated knock-down of *Drosophila* RCC1, either in the presence or absence of centrosomes, has led some to propose that the Ran.GTP gradient is unnecessary in flies (Moutinho-Pereira et al., 2013). Furthermore, there is no apparent homolog of RanBP1, which is essential for Ran function in human cells (Bischoff and Görlich, 1997; Floer et al., 1997; Fornerod and Ohno, 2002; Kuersten et al., 2002; Seewald et al., 2003), in *Drosophila*. Finally, whereas RanGAP localizes to spindles and kinetochores in mammalian cells (Joseph et al., 2002), in *Drosophila* early embryos it is predominantly found at the nuclear envelope, and occasionally at peripheral spindle MTs (Trieselmann and Wilde, 2002).

However, these apparent differences need not preclude a functional role for Ran.GTP in *Drosophila* spindle formation. The absence of an RNAi-induced RCC1 phenotype can be explained by incomplete knockdown. Indeed, although RCC1 knockdown in HeLa cells has been shown to result in reduced Ran.GTP generation around chromatin, it has no effect on mitotic spindle assembly either (Bamba et al., 2002; Kaláb et al., 2006). Similarly, the differences in localization of Ran pathway components between *Drosophila* and other cells may reflect detection issues or system specialisms. For example, given the syncytial nature of the *Drosophila* early embryo, the presence of RanGAP at the nuclear envelope may in fact, create an effective barrier, which restricts Ran-mediated spindle assembly around each individual mass of chromatin, rather than allowing diffusion throughout the entire embryo. Moreover, a biochemical association between Ran and MTs has been demonstrated by mass spectrometry (Hughes et al., 2008), suggesting a functional relationship between the Ran pathway and spindle formation.

Until very recently, the strongest evidence supporting a role for Ran in *Drosophila* mitosis came from studies by the Wilde lab (Trieselmann and Wilde, 2002; Silverman-Gavrila and Wilde, 2006). Microinjecting syncytial embryos with dominant negative Ran (T24N) resulted in a range of phenotypes ranging from the severe, such as complete failure to generate MTs or very few MTs, to more moderate, such as spindle fusion and spindle pole disorganization. Moreover, injection of Ran inhibitors such as Importin α/β and human RanBP1 reproduced these less severe phenotypes. These results indicate that the Ran pathway does have a MT-related role in the embryo (Silverman-Gavrila and Wilde, 2006). However, the dominance of centrosome-driven spindle formation in this tissue made it difficult to address the precise role of Ran in generation of MTs from chromatin in *Drosophila*. Recently, this barrier has been overcome by exploiting the temperature dependent nature of MT polymerization. When *Drosophila* embryos that have built a bipolar spindle using centrosomes are cooled to 4°C, their MTs depolymerize. Upon return to room temperature, a dramatic shift in the temporal and spatial nucleation, stabilization and sorting of MTs occurs (Hayward et al., 2014); instead of MTs re-initiating from the centrosomes, the mitotic spindle is organized almost exclusively from MTs generated around chromosomes (Hayward and Wakefield, 2014; Hayward et al., 2014). When dominant negative Ran (T24N) is injected into these cold-treated *Drosophila* embryos and spindle formation re-initiated following the temperature shift, chromatin-dependent MT generation is completely abolished (Hayward and Wakefield, 2014). Thus, it seems highly likely that the Ran pathway does function in *Drosophila* similarly to vertebrate systems.

### The role of karyopherins (importins and exportins) in *Drosophila* mitosis

Ran.GTP largely elicits its cellular functions through affecting the interactions between a class of proteins, termed Karyopherins, and their targets (Chook and Blobel, 2001; Mosammaparast and Pemberton, 2004). Karyopherins can be broadly divided into two classes—those involved in nuclear import (Importins), and those involved in nuclear export (Exportins). While Exportins bind Nuclear Export Signals (NESs), Importins bind Nuclear Localization Signals (NLSs). Together, these proteins therefore coordinate the transport of cargo in and out of nucleus (Figure 2). A sub-set of NLS-containing Importin cargoes, those that affect MT function during mitosis, are termed SAFs. During interphase these SAFs are sequestered in the nucleus by Importins, preventing interaction with interphase MTs. However, during open mitosis, the nuclear envelope breaks down, resulting in an influx of Tubulin dimer to the nucleus. The SAFs are then able to interact with Tubulin or with nascent MTs nucleated in the nuclear space, promoting spindle assembly (Figure 1B).

The complex regulation of Ran-mediated Importin-SAF interactions during both interphase and mitosis is derived from the specificity of the NLS-Importin interaction. The most well characterized example is the Importin β, Karyopherin β1, which can recognize targets either independently (Palmeri and Malim, 1999) or in association with an adapter protein—one of several variants of Importin α (Goldfarb et al., 2004). Each Importin α variant imparts specificity for different subsets of cargo (Pumroy and Cingolani, 2015). Moreover, many other variants of Karyopherin β exist, which recognize different NLSs and do not require an Importin α adaptor (Chook and Süel, 2011). Karyopherin β2 is one such example, which has also been implicated in mitotic regulation, through recognition of distinct NLS motifs (Lau et al., 2009; Bernis et al., 2014).

In all, over 20 different Karyopherins exist, with the vast majority having *Drosophila* homologs (Quan et al., 2008; Table 1). Even though only partial nuclear envelope breakdown occurs during most *Drosophila* mitoses, (Fuchs et al., 1983; Stafstrom and Staehelin, 1984; Harel et al., 1989; Katsani et al., 2008), the importance of nuclear import/export of proteins on mitotic spindle formation is clear. Abrogation of Importin α/β binding to NLSs, through injection of NLS peptides, results in monopolar, unfocused, narrow, and large barrel-shaped spindles, while co-injection of NLS peptide with dominant-negative Ran rescues spindle assembly, confirming that these phenotypes are due to Importin binding to NLSs (Virágh et al., 2012).

**Table 1:** **List of commonly described karyopherins and their ***Drosophila*** homologs**.

**Vertebrate name**	***Drosophila* homolog**	***Drosophila* ID**
**KARYOPHERIN/IMPORTIN α FAMILY**		
Karyopherin α1/importin α5	Karyopherin α1	CG8548
Karyopherin α5/importin α6		
Karyopherin α6/importin α7		
Karyopherin α2/importin α1	Karyopherin α2/Pendulin	CG4799
Karyopherin α3/importin α4	Karyopherin α3	CG9423
Karyopherin α4/importin α3		
Not identified	Karyopherin α4	CG10478
**KARYOPHERIN β FAMILY**		
Karyopherin β1/Importin β1	Female sterile(2) Ketel/Importin β	CG2637
Karyopherin β2/Transportin1 & 2 (TNPO1, 2)	Transportin	CG7398
	CG8219	CG8219
Transportin 3 (TNPO3)	Transportin Serine/Arginine Rich	CG2848
Karyopherin β3/Importin 5/RanBP5 (IPO5)	Karyopherin β3	CG1059
Importin 4/RanBP4	CG32164	CG32164
	CG32165	CG32165
Importin 7/RanBP7	Moleskin	CG7935
Importin 8/RanBP8		
Importin 9	RanBP9	CG5252
Importin 11	RanBP11	CG33139
Importin 13	Cadmus	CG7212
Exportin 1/Crm1	Embargoed/Crm1	CG13387
Exportin 2/Cas/CSE1L	Cas	CG13281
Exportin 4	Not identified	
Exportin 5	RanBP21	CG12234
Exportin 6	Ellipsoid body open/Exp6	CG3923
Exportin 7/RanBP16	RanBP16	CG33180
Exportin-tRNA/XPOT	Not identified	

*The list outlines the Drosophila homologs of commonly described vertebrate karyopherins. Karyopherin α family members cannot bind Ran.GTP directly, relying on Importin β. In general, Ran-dependent SAFs are inhibited by Importin α/β dimer. The evolutionarily relationships between karyopherins is complex, and homologs have been identified through bioinformatics methods (Quan et al., 2008; Mason et al., 2009; Chook and Süel, 2011). While their discussion is beyond the scope of this review, all karyopherins have the potential to have a role in the Ran-mediated spindle assembly pathway*.

The most widely studied Importin in flies, Importin β1/Ketel, localizes predominantly to the nuclear envelope throughout the cell cycle of the early embryo (Trieselmann and Wilde, 2002) and, biochemically, is able to bind MTs (Tirian et al., 2003; Hughes et al., 2008). However, a point mutation which locks Importin β1/Ketel in a Ran.GDP binding conformation has no effect on nuclear import or spindle formation, though it does affect nuclear envelope reformation (Timinszky et al., 2002), suggesting other Importin β proteins contribute to SAF release during mitosis. Indeed, Importin 5/Karyβ3 has been identified as a MT-associated protein (Hughes et al., 2008) and as an interactor of the SAF *Drosophila* Hepatoma Up-Regulated Protein (dHURP), alongside Importin β1/Ketel (Hayward and Wakefield, 2014). Thus, it is conceivable that Importin 5/Karyβ3 may sequester a specific subset of SAFs that are involved in embryonic spindle formation. Of the other Karyopherin β or α proteins present in *Drosophila*, nothing is known with regards to potential mitotic SAF-related roles. Indeed, even in other experimental systems, the vast potential array of Karyopherin combinations currently precludes a comprehensive understanding of the role of Importin-SAF release on mitotic spindle formation.

## Ran-dependent SAFs

The list of Ran-dependent SAFs has steadily increased since the first targets, TPX2 and NuMA, were identified in 2001 (Gruss et al., 2001; Wiese et al., 2001). These SAFs, primarily characterized in humans and *Xenopus*, play diverse roles and contribute to many aspects of spindle formation and function. Unsurprisingly, given the localization of Ran.GTP around chromatin, the majority of these have key roles in chromosome-mediated spindle self-assembly. However, several also function in the nucleus during interphase, including TPX2 (Neumayer and Nguyen, 2014), CHD4 (O'shaughnessy and Hendrich, 2013), NuMA (Ohata et al., 2013; Vidi et al., 2014), ISWI (Aydin et al., 2014; Vidi et al., 2014), Bard1 (Jasin, 2002), NuSAP (Kotian et al., 2014), Adenomatous Polyposis Coli (Apc) (Jaiswal and Narayan, 2008), TACC3 (Ha et al., 2015), and Survivin (Chakravarti et al., 2004; Jiang et al., 2009; Reichert et al., 2011); all of which have been implicated in the DNA damage response. Of the 29 Ran-dependent SAFs considered here, 25 have annotated homologs in *Drosophila* (Table 2). Many of these have cellular roles related to that of their vertebrate counterparts, and yet very few have been functionally verified as being Ran dependent. It is therefore likely that the overarching biochemical pathways and processes of Ran-mediated spindle assembly are conserved, though with some re-wiring unique to *Drosophila*. For the purposes of this review, we have separated SAFs into two groups, motor (Table 2) and non-motor SAFs (Table 3). In the following sections, we summarize the known functions of these proteins in Ran-dependent mitotic pathways and compare their roles in *Drosophila* and non-*Drosophila* models.

**Table 2:** **Comparison of ***Drosophila*** Kinesins identified as mitotically relevant and their human homologs**.

***Drosophila* name**	***Drosophila* ID**	**Interphase localization (*Drosophila*)**	**Mammalian name**	**Interphase localization (mammalian)**	**Verified as Ran-mediated?**
Klp61F	CG9191	MTs	Eg5/KIF11	Diffuse	In *Drosophila*
Klp10A	CG1453	MTs	KIF2A/B/C	MT plus-ends	Not studied
Klp67A	CG10923	Nucleus	Kip3/KIF18A/B	Nucleus	Not studied
Ncd	CG7831	Nucleus	KIFC1	Nucleus	In *Xenopus*
CENP-Meta	CG6392	Kinetochore	CENP-E	Diffuse	Not studied
Klp3A	CG8590	Nucleus	KIF4	Nucleus and MT plus-ends	In *Drosophila*
Nod	CG1763	Nucleus and MT plus-end	Kid/KIF22	Nucleus	In human cells
Pavarotti	CG1258	Nucleus	MKLP1/KIF23	Nucleus	Not studied
Subito	CG12298	Nucleus	MKLP2/KIF20A	Nucleus	Unaffected in *Drosophila*

*The table compares the interphase localization of Klp61F^1^ to Eg5^2^, Klp10A^1^ to KIF2^2^, Klp67A^1^ to KIF18^2^, Ncd^1^ to KIFC1^2^, CENP-meta to CENP-E (Schaar et al., 1997; Yucel et al., 2000), Klp3A to KIF4 (Williams et al., 1995; Morris et al., 2014), Nod to Kid (Levesque and Compton, 2001; Cui and Hawley, 2005), Pavarotti^1^ to MKLP1^2^, and Subito to MKLP2^2^. Aside from CENP-meta/CENP-E, all other kinesins have similar interphase localization. To date, only four kinesins have been verified to be directly affected by Ran*.

1 *Goshima and Vale, 2005*.

2 *Syred et al., 2013*.

**Table 3:** **List of non-motor SAFs and a summary of their functions**.

**Vertebrate name**	***Drosophila* name**	***Drosophila* ID**	**Function during vertebrate mitosis**
CHD4	MI-2	CG8103	Stabilizes MTs^*^
NuMA	Mud	CG12047	Anchors MTs to cell cortex^*^
	Asp	CG6875	Focuses spindle poles^*^
ISWI (SMARCA5)	ISWI	CG8625	Nucleates and bundles MTs^*^
Bard1	None	None	Localizes TPX2 to spindle poles
RHAMM (HMMR)	None	None	Facilitates centrosomal MT nucleation
HURP (DLGAP5)	Mars/D-HURP	CG17064	Facilitates both centrosomal and chromosomal MT generation and MT-kinetochore attachment, nucleate and stabilize MTs^*^
NuSAP1	Mink	CG11120	Targets crosslinked MTs to chromatin^*^
TPX2	Mei38/dTPX2	CG14781	Bundles MTs, facilitates Aurora A function, facilitates chromatin-driven MT generation^**^
Mel28/ELYS (AHCTF1)	CG14215	CG14215	Recruits Nup107–160 complex to the kinetochore
Rae1	Rae1	CG9862	Increases ability of NuMA to attract MTs
APC	APC	CG1451	Anchors MTs to cell cortex, focus spindle poles
TACC3	dTACC	CG9765	Promotes MT polymerization, MT-kinetochore attachment^*^
MCRS1	Rcd5	CG1135	Protects MT from depolymerization
AKAP450 (AKAP9)	dPlp	CG33957	Recruit Ran.GTP to the centrosome
Xnf7	None	None	Bundles MTs and protects against depolymerization
Crumbs3 (CRB3)	Crumbs	CG6383	Centrosomal regulation
Npm1	Nph	CG7911	Facilitates centrosome duplication
Survivin (BIRC5)	Deterin	CG12265	Loads TPX2 onto MTs
Lamin B1 (LMNB1)	Lamin	CG6944	Acts within the spindle matrix
CDK11	Pitslre	CG4268	Kinase which regulate centrosome maturation and separation, as well as chromatid cohesion
RanGAP1	RanGAP/Sd	CG9999	Negatively regulates Ran through activating GTPase activity^*^
RanBP1	None	None	Negatively regulates Ran through activating GTPase activity
RanBP2	Nup358	CG11856	Negatively regulates Ran through activating GTPase activity

*Most Ran-dependent SAFs have homologs in Drosophila*.

* *Asterisks denote Drosophila proteins that have similar reported mitotic functions to vertebrate counterparts, although most have not been characterized as Ran-dependent*.

** *dTPX2 bundles MTs and stabilizes chromatin-induced spindle formation, but lacks Aurora A regulating activity*.

### Non-motor SAFs

Although TPX2 was one of the first Ran-dependent SAFs to be identified, examination of the *Drosophila* genome by standard homology-based methods, over a period of a decade failed to identify a TPX2 homolog. More recent analysis found that the protein encoded by the mei38/short spindles 1 (ssp1) locus shares significant homology to parts of the human TPX2 protein, though lacking both the Aurora A and Kinesin-5 binding domains (Goshima, 2011; Hayward et al., 2014). Based on current Flybase nomenclature, we will refer to this protein as Mei38/dTPX2, but acknowledge the uncertainty that comes with attributing standardized names to divergent gene products from different species. In vertebrates, TPX2 has multiple mitotic roles; it can directly nucleate and bundle MTs (Brunet et al., 2004), it facilitates autophosphorylation of the mitotic kinase Aurora A and protects it from dephosphorylation (Dodson and Bayliss, 2012; Zorba et al., 2014), and it targets Xklp2/kinesin-12/KIF15 to spindle poles (Wittmann et al., 1998). TPX2 is also required for localization of KIF11/Eg5 to spindle MTs and facilitates its functions in kinetochore MT formation and spindle pole organization (Ma et al., 2011). In contrast, Mei38/dTPX2 does not bind Aurora A and a *mei38* null mutation does not affect Aurora A or the localization of its effectors in *Drosophila* embryos (Hayward et al., 2014). Moreover, as *Drosophila* lack a clear Xklp2/kinesin-12/KIF15 homolog (Wickstead et al., 2010), and as Mei38/dTPX2 lacks a kinesin-5 binding domain (Goshima, 2011), it appears likely that Mei38/dTPX2 does not possess kinesin-directed activities. However, Mei38/dTPX2 has MT bundling capacity *in vitro* (Goshima, 2011) and mitotic spindles lacking Mei38/dTPX2 are significantly shorter than wild type (Hayward et al., 2014; Helmke and Heald, 2014; Fu et al., 2015), suggesting that at least some MT-related functions of vertebrate TPX2 (Hayward et al., 2014; Helmke and Heald, 2014; Fu et al., 2015) are shared with Mei38/dTPX2. Indeed, although *Drosophila* lacking Mei38/dTPX2 are viable and fertile, the protein does have a crucial role during self-organization of the mitotic spindle. Upon chromatin-derived spindle self-assembly driven by cold treatment and regrowth, the *Drosophila* embryonic spindles are unstable and tend toward collapse (Hayward et al., 2014). Taken together, the current evidence suggests that Mei38 may well represent a divergent TPX2-like protein, where additional proteins (e.g., see Section Ran.GTP and Mitotic Kinases) compensate for the loss of the key functions maintained in both *Xenopus* and humans.

Similarly, there is no true *Drosophila* homolog of the conserved SAF, Nuclear Mitotic Apparatus (NuMA). NuMA was initially described as an Importin β-inhibited protein promoting MT aster formation (Wiese et al., 2001) and has two clear roles during spindle formation. First, it is transported to MT minus-ends by the Dynein-Dynactin complex, where it crosslinks MTs and focuses the spindle poles (Gaglio et al., 1995; Merdes et al., 1996, 2000; Compton, 1998; Radulescu and Cleveland, 2010). Second, it plays a role in anchoring of astral MTs to the cell cortex, correctly orienting mitotic spindles during both symmetric (Silk et al., 2009; Kotak et al., 2012) and asymmetric cell division (Morin and Bellaïche, 2011; Peyre et al., 2011). In *Drosophila*, these two functions appear to be carried out by two distinct proteins. The Mushroom Body Defect (Mud) protein is the closest homolog to NuMA as assessed by primary structural homology (Bowman et al., 2006), while the Abnormal Spindle protein (Asp) also possesses some limited homology to NuMA domains (Saunders et al., 1997). Both proteins localize to the spindle poles during syncytial mitosis (Saunders et al., 1997; Yu et al., 2006). However, while Mud does not appear to have a role in focusing spindle poles, the absence of Asp leads to splayed spindle poles, free centrosomes and downstream mitotic and meiotic abnormalities (do Carmo Avides et al., 2001; Wakefield et al., 2001). Conversely, loss of Mud specifically affects MT-cortex interactions (Bowman et al., 2006; Izumi et al., 2006; Siller et al., 2006). In vertebrates, the membrane-anchored heterotrimeric G-proteins (mainly Gαi1, Gαi2, and Gαi3) interact with the leucine-glycine-asparagine repeat protein (Lgn) (Siderovski et al., 1999), which in turn interacts with NuMA and, though this, Dynein to tether MTs (Du et al., 2001; Bergstralh and St Johnston, 2014). This NuMA-facilitated anchoring is inhibited in the central region of the cell surrounding the metaphase plate, where the concentration of Ran.GTP is high (Kiyomitsu and Cheeseman, 2012; Bird et al., 2013), and as such is key to the orientation of the bipolar spindle (Morin et al., 2007; Konno et al., 2008; Zheng et al., 2010). In *Drosophila*, Mud binds to the homolog of Lgn, Partner of Inscrutable (Pins), and Gαi1 (Siller et al., 2006). The requirement of Ran in Mud function is indirect. Targeting of Mud and Pins to the cortex requires an additional protein, Canoe/Afadin (Wee et al., 2011), and only occurs when Canoe is bound to Ran.GTP; it is theorized that Canoe brings Ran.GTP to the cortex, and the localized high concentration of cortical Ran.GTP prevents Importin β from inhibiting Mud (Wee et al., 2011). This is in sharp contrast to the situation in vertebrates, where NuMA-mediated anchoring is inhibited by Ran.GTP (Kiyomitsu and Cheeseman, 2012; Bird et al., 2013); this discrepancy outlines the incompleteness of our knowledge of Ran function in spindle pole positioning.

Hepatoma Up-Regulated Protein (HURP) is a MT nucleating and stabilizing SAF, important in both centrosome and chromatin driven spindle assembly (Koffa et al., 2006). Crucially, its *Drosophila* homolog, Mars/dHURP, is one of the few *Drosophila* SAFs for which there is evidence for a Ran-dependent function (Cesario and McKim, 2011). In vertebrates, HURP plays a crucial role in kinetochore-fiber stabilization (Koffa et al., 2006; Sillje et al., 2006; Wong and Fang, 2006). During mitosis HURP is phosphorylated by Aurora A, which protects it from degradation (Yu et al., 2005), allowing its association with MT plus-ends and facilitating kinetochore-MT attachments (Wu et al., 2013). While the dependence of dHURP by Aurora A has not yet been investigated, its role in kinetochore-fiber stabilization and kinetochore-MT attachment appears to be conserved (Yang and Fan, 2008). dHURP is also required for MT attachment to centrosomes (Zhang et al., 2009) and for localization of γ-tubulin to the spindle (Yang and Fan, 2008), loss of which results in decreased spindle density. Moreover, in both *Drosophila* and vertebrate systems, HURP has been shown to be critical for chromatin-mediated MT generation (Wong and Fang, 2006; Hayward et al., 2014). Therefore, while certain functions have not yet been verified between vertebrate and *Drosophila* systems, dHURP is an excellent candidate for further studies of Ran-mediated spindle assembly in *Drosophila*.

The Transforming Acidic Coiled Coil (TACC) family of proteins, which includes TACC3 in humans, exist in a complex with TOG/XMAP215 to promote MT polymerization (Fox et al., 2014; Gutiérrez-Caballero et al., 2015), and Clathrin to promote kinetochore-MT bundling (Fu et al., 2010; Hubner et al., 2010; Lin et al., 2010; Booth et al., 2011). It therefore stabilizes kinetochore MTs (Booth et al., 2011) centrosomal MTs (Barros et al., 2005) and the mitotic spindle, generally (Gergely et al., 2000a). Although not commonly regarded as a Ran-dependent SAF, TACC3 immunoprecipitates with Importin β, and their interaction is reduced in the presence of constitutively active RanL43E (Albee et al., 2006); further, in human cell lines, TACC3 is sequestered to the nucleus during interphase, presumably by Importins (Gergely et al., 2000a). In flies, the interaction between the TACC3 and TOG homologs (dTACC and Msps respectively) is conserved. dTACC, however, lacks an apparent NLS and does not localize to the nucleus, but concentrates at centrosomes during embryonic interphase (Gergely et al., 2000b). This might indicate that dTACC is not Importin-mediated. However, dTACC localization is disrupted in the presence of dominant negative Ran (T24N) during *Drosophila* female meiosis (Cesario and McKim, 2011). Since TACC3 and dTACC both localize to spindle MTs during mitosis (Barros et al., 2005; Kinoshita et al., 2005), and are functionally conserved (Gergely et al., 2000a), *Drosophila* may be a useful tool to explore the mechanistic details between Ran and TACC.

During interphase, the Nucleolar and Spindle Associated Protein (NuSAP) plays a role in the DNA damage response (Kotian et al., 2014). However, at the onset of mitosis, NuSAP is released around chromatin by Ran.GTP, binding to DNA and both stabilizing and crosslinking MTs in order to promote spindle formation from the chromosomes (Raemaekers et al., 2003; Ribbeck et al., 2006, 2007). Cells either deficient in or overexpressing NuSAP show defects in cell division (Raemaekers et al., 2003; Ribbeck et al., 2006; Li et al., 2007) and knockout of NuSAP in mice is lethal at the early embryonic stage (Vanden Bosch et al., 2010). The *Drosophila* homolog of NuSAP, Mink, was recently identified as a MAP in mitotic, but not interphase, S2 cells (Syred et al., 2013). While the role of Ran in Mink function has not yet been investigated, it is nuclear during interphase and therefore is likely to be bound by Importins, and has a role in crosslinking and stabilizing MTs in *Drosophila* S2 cells (Syred et al., 2013).

Somewhat surprisingly, three nucleic acid binding proteins have been identified as Ran-dependent SAFs. The chromatin remodeling factors, Imitation Switch (ISWI) and Chromodomain-Helicase-DNA-binding 4 (CHD4), both associate with MTs in a Ran.GTP-dependent manner in *Xenopus* (Yokoyama et al., 2009, 2013). Both localize to chromatin during interphase but relocalize to the spindle during mitosis in *Xenopus* egg extracts, and both play roles in spindle assembly and stability. ISWI can nucleate and bundle MTs in *Xenopus* embryo extracts (Yokoyama et al., 2009), while CHD4 stabilizes, but does not nucleate, MTs (Yokoyama et al., 2013). Encouragingly, both have *Drosophila* homologs which appear to function similarly to their vertebrate counterparts (Yokoyama et al., 2009, 2013). CHD4 acts in early mitosis to promote spindle assembly from the chromatin, and depletion in either HeLa cells or *Drosophila* S2 cells results in reduced spindle density and disorganization, with resultant chromosome mis-segregation (Yokoyama et al., 2013). ISWI stabilizes the spindle prior to anaphase, and depletion of this protein in *Xenopus* egg extracts or *Drosophila* S2 cells produces defective and disintegrating spindles (Yokoyama et al., 2009). In addition, Rae1, an mRNA export protein normally found in the nucleus, binds directly to Importin β (Blower et al., 2005), suggesting that its activity is spatially and temporally coordinated by Ran. Indeed, it appears to act by increasing the ability of NuMA to focus and bundle MTs and perturbation of this interaction results in spindle defects (Wong et al., 2006). Interestingly, Rae1 acts as part of a large ribonucleoprotein complex, which includes Nup98 and the Ran-dependent SAF, TACC3 (Blower et al., 2005), though the modes of activation and regulation of this complex are unclear. However, as yet, nothing is known of Rae1 mitotic MT function in *Drosophila*.

Two additional MT effectors, APC and Microspherule Protein 1 (MCRS1) have been identified as Ran-dependent SAFs in vertebrates (Dikovskaya et al., 2010; Meunier and Vernos, 2011), and have *Drosophila* homologs. APC is involved in a multitude of cellular functions, including Wnt signaling, transcription, DNA damage response, cell adhesion, and mitosis (Bahmanyar et al., 2009; Lui et al., 2012). In both *Drosophila* and vertebrates, there are two APC genes; *apc* and *apc2*. In vertebrates, loss of APC results in embryonic lethality, while APC2 knock-outs are viable. In *Drosophila*, APC and APC2 appear to function redundantly in Wnt/Wingless signaling though the distribution of each differs in *Drosophila* tissues (Ahmed et al., 2002). APC has multiple mitotic roles in vertebrates. It can bind either directly or indirectly to MTs and plays roles in nucleation, bundling, and MT dynamics (Deka et al., 1998; Banks and Heald, 2004; Dikovskaya et al., 2004), affecting processes such as kinetochore attachment and MT anchoring at the centrosomes (Bahmanyar et al., 2009). However, in *Drosophila*, it is APC2 that appears to play a more crucial role in mitosis. Early embryos derived from hypomorphic *apc2* alleles show defects in centrosome separation and spindle pole positioning and subsequent loss of nuclei from the embryonic cortex in a process called “nuclear fall-out” (McCartney et al., 2001; Buttrick et al., 2008). This is thought to occur through loss of stable attachments between astral microtubules plus ends to the cell cortex, a process mediated via catenins (McCartney et al., 2001; Buttrick et al., 2008). Moreover, RNAi of apc2 in *Drosophila* also interferes with oriented cell division in the embryonic epidermis, suggesting a wider role for APC2 in regulating MT-cortex interactions (Lu et al., 2001). It is unclear whether this function is conserved in vertebrate APC or APC2, though APC loss in mammalian cell lines results in mis-orientation of the spindle (Green et al., 2005). In vertebrates, APC protein function is mediated by Ran; vertebrate APC interacts with Importin β, and this interaction is reduced by constitutively-active Ran-Q69L (Dikovskaya et al., 2010). Further, Importin β inhibits the ability of APC to nucleate MTs from pure Tubulin, and to bundle Taxol stabilized MTs (Dikovskaya et al., 2010). However, whether Ran also regulates *Drosophila* APC or APC2 function is as yet unknown. Since *apc2* mutant embryos present such a distinctive centrosome and cortical phenotype in the *Drosophila* syncytial embryo (see above), inhibition of Ran function in these mutants, under both normal cycling and cold-treatment/re-growth conditions, presents an ideal opportunity to test this hypothesis. Much less is known about the *Drosophila* homolog of MCRS1. In vertebrates, MCRS1 plays a protective role at the minus ends of MTs generated from the chromosomes, preventing depolymerization (Meunier and Vernos, 2011). Interestingly, although the *Drosophila* homolog, Rcd5, has been implicated in transcription during interphase (Andersen et al., 2010), it was originally identified in a genome-wide screen for proteins that affected the recruitment of the MT effector, Centrosomin (Cnn), to centrosomes in mitotic S2 cells—hence its name, Reduction in Cnn dots 5 (Dobbelaere et al., 2008). However, it remains unclear whether Rcd5 has a mitotic role, or whether it is Ran-regulated.

One further Ran-dependent SAF with a sequence homolog in *Drosophila*, is the cell polarity protein Crumbs3 (Crumbs in *Drosophila*) (Pocha and Knust, 2013). Although generally regarded as a transmembrane protein, the role of Crumbs3 in mitosis is dependent upon a specific splice variant with a distinct C-terminus, Crumbs3-CLPI. Crumbs3-CLPI appears to play a key role in centrosomal regulation, and depletion in mammalian cells leads to a variety of phenotypes including supernumerary centrosomes, multipolar spindle formation and multinuclear cells (Fan et al., 2007). Crumbs3-CLPI is dependent upon Ran.GTP for localization to centrosomes (Fan et al., 2007), and may be recruited by a centrosomal pool of Ran.GTP. However, the *Drosophila* genome does not appear to possess such a distinct splice variant and so whether Crumbs is able to moonlight as a Ran effector in flies is currently unknown.

Finally, there are also three Ran-dependent SAFs identified in vertebrates that appear to have no sequence homologs in *Drosophila*. These are Bard1, which associates with BRCA1 to localize TPX2 to spindle poles (Joukov et al., 2006); RHAMM, which interacts with TPX2 and γ-Tubulin at centrosomes (Groen et al., 2004); and Xnf7, which bundles MT and protects them from depolymerization (Maresca et al., 2005). These proteins feature domains and coiled-coil motifs that confound conventional search methods, and as such it is difficult to determine whether they are indeed absent in *Drosophila* or still remain to be identified. Bard1, at least, has been identified in the genome of the honeybee *Apis mellifera* and therefore may have been lost or truncated in *Drosophila* and other Diptera, similarly to dTPX2/Mei38. Alternatively, RHAMM and Xnf7 may represent vertebrate-specific proteins; analysis of the evolutionary patterns of these proteins across a range of opisthokont organisms would answer this question.

### Motor SAFs

MT motor proteins play essential roles in regulating MT dynamics and generating the forces involved in centrosome movement, and chromosome alignment and segregation. The minus-end directed motor cytoplasmic Dynein is important throughout mitosis, with roles in centrosome separation (Vaisberg et al., 1993; Robinson et al., 1999; Raaijmakers et al., 2012), spindle pole focusing in conjunction with NuMA (Radulescu and Cleveland, 2010), and shedding of the Rod-Zw10-Zwilch (RZZ) complex from kinetochores polewards along kinetochore-MT bundles (Barisic and Geley, 2011). Kinesin-like proteins (Klps) include both plus end- and minus end- directed motors which can transport cargo to specific cellular locations, crosslink and slide MTs to generate forces for centrosome or chromosome separation, attach MTs to kinetochores, push MTs away from chromatin, or alter MT dynamics; see Cross and McAinsh (2014) for a recent review.

Of the 25 kinesins identified in *Drosophila*, depletion of 9 results in mitotic defects (Table 2; Yucel et al., 2000; Giunta et al., 2002; Goshima and Vale, 2005; Cesario et al., 2006). These 9 mitotically relevant kinesins have homologs in humans and five—Klp3A (KIF4), Klp67A (KIF18), Ncd (KIFC1), Pavarotti (KIF23), and Nod (Kid/KIF22)—are sequestered to the nucleus in both *Drosophila* interphase cells (Cui et al., 2005; Goshima and Vale, 2005) and human cell lines (Mazumdar et al., 2011; Syred et al., 2013). Expression of Klp67A, Ncd, or Pavarotti without their NLS results in MT network disruption (Goshima and Vale, 2005), highlighting the importance of sequestering them away from interphase MTs. However, whether Ran plays a role in the mitotic function of these kinesins beyond sequestration during interphase in *Drosophila* is largely unknown.

Ran has, however, been shown to regulate two kinesins in *Drosophila*; Klp61F (KIF11/Kinesin-5/Eg5) and Klp3A (Kinesin-4/KIF4), though it is not clear how this impacts upon spindle assembly. Klp61F shows reduced binding affinity to MTs in the presence of dominant negative Ran (T24N) (Silverman-Gavrila and Wilde, 2006), a property that is conserved in its *Xenopus* homolog, Eg5 (Wilde et al., 2001). Notably, in both *Drosophila* and vertebrates, Klp61F/KIF11 is excluded from the nucleus during interphase, indicating that Ran is able to mediate the protein's activity in the absence of an NLS through an unknown mechanism (Silverman-Gavrila and Wilde, 2006). Less is known about Klp3A, though upon injection of dominant negative Ran (T24N) into *Drosophila* syncytial embryos, Klp3A localization to MTs is lost, and instead re-localizes to chromosomes (Silverman-Gavrila and Wilde, 2006).

Outside of *Drosophila*, Ran has been shown to affect the regulation of the minus-end directed motor KIFC1 (also known as HSET or XCTK2) (Ems-McClung et al., 2004; Weaver et al., 2015), and the chromokinesin, Kid (KIF22) (Trieselmann et al., 2003). Importin α/β binds the non-motor tail of KIFC1 and inhibits its MT crosslinking activity (Ems-McClung et al., 2004). Therefore, the Ran.GTP gradient effectively limits the MT sliding activity of KIFC1 to the regions near the chromosomes (Weaver et al., 2015); indeed, expressing mutant forms of KIFC1 which cannot be inhibited by Importin α/β causes spindles to become elongated (Cai et al., 2009). The chromokinesin Kid/KIF22 plays a key role in generating the forces that align and maintain chromosomes at the metaphase plate (Antonio et al., 2000; Funabiki and Murray, 2000; Levesque and Compton, 2001). This activity relies upon Kid's motor functions, but Importin α/β is able to directly inhibit its MT binding ability (Trieselmann et al., 2003). This inhibition of MT binding ability effectively targets Kid to chromosomes, where Kid is released from Importin α/β in the presence of high Ran.GTP (Tahara et al., 2008). While *Drosophila* has homologs of both KIF1C and Kid, Ncd and Nod respectively, the involvement of Ran in the function of these proteins has not yet been determined.

### Ran.GTP and mitotic kinases

Kinases play a central role in mitosis, from cell cycle control to spindle assembly, and in cytokinesis. The classic mitotic kinases include the Cyclin Dependent Kinase, CDK1, the Polo-like kinases (PLKs), the Aurora kinases A and B, and the NIMA (never in mitosis in *Aspergillus nidulans*)-related kinases (NEKs) (Ma and Poon, 2011; Bayliss et al., 2012). It is therefore unsurprising that at least some of these kinases are regulated by, or themselves regulate, Ran. To date, there is evidence linking CDK1, Plk1, and Aurora A to Ran-dependent pathways in mitosis.

CDK1 is the master regulator of mitotic entry (Malumbres, 2014), and plays a role in Ran-mediated spindle assembly by phosphorylating RCC1 (Hutchins et al., 2004). RCC1 contains an N-terminal NLS, which mediates nuclear localization (Nemergut and Macara, 2000; Talcott and Moore, 2000). During interphase, the interaction of RCC1 with chromatin is highly dynamic, cycling between chromatin and nucleoplasm; the interaction of RCC1 with chromatin is coupled to its ability to phosphorylate Ran and phosphorylation dissociates RCC1 from chromatin (Li et al., 2003). During mitosis, phosphorylation of the NLS of RCC1 by CDK1 is required to maintain the dynamic nature of this RCC1-chromatin interaction, preventing RCC1 inhibition by Importins (Hutchins et al., 2004; Li and Zheng, 2004). This phosphorylation event is therefore essential for generation of the Ran.GTP gradient around the chromatin, and disruption results in abnormal spindle formation and chromosome mis-segregation (Li and Zheng, 2004). Again, there are unfortunately no published studies investigating the relationship between *Drosophila* CDK1 and Ran pathway components.

Plk1 regulates, amongst other things, mitotic entry, centrosome maturation, spindle assembly, and cytokinesis (Zitouni et al., 2014). During interphase in human cells, Plk1 localizes to both the cytoplasm and the nucleus, though disruption of the NLS blocks mitotic entry, suggesting constant shuttling in and out of the nucleus impacts cell cycle progression (Taniguchi et al., 2002). During mitosis, Plk1 phosphorylates Ran, and while the precise functional significance of this is unknown, a Plk1 phospho-mimetic mutation in Ran results in abnormal spindle morphology (Feng et al., 2006). Plk1 also phosphorylates RanBP1 (Hwang et al., 2011), and this phosphorylation is crucial for proper spatial regulation of the Ran.GTP gradient (Zhang et al., 2014). Together, this suggests that Plk1 functions upstream of Ran-dependent mitotic pathways. However, nothing is yet known of the relationship between the *Drosophila* homolog of Plk1, Polo, and Ran.

There is, however, some evidence linking Aurora kinases and Ran in *Drosophila*. In vertebrate systems, the Ran-dependent SAF, TPX2, is at the heart of Aurora A localization and activation. However, as described in Section Non-motor SAFs, *Drosophila* TPX2 (Mei38) lacks the Aurora A interaction motif and Aurora A does not bind to dTPX2 (Goshima, 2011; Hayward et al., 2014), neither does loss of the protein affect the localization of Aurora A effectors (Hayward et al., 2014). However, injection of dominant negative Ran into *Drosophila* early embryos inhibits both the centrosomal and spindle association of Aurora A, demonstrating a clear regulatory link between the two (Silverman-Gavrila and Wilde, 2006). How this is achieved is unclear, but there are two potential pathways. Firstly, HURP plays a role in Aurora A targeting in *Xenopus* (Koffa et al., 2006). In *Drosophila* dHURP has been demonstrated to be regulated by Ran.GTP (Cesario and McKim, 2011), and loss of either dHURP or injection of dominant negative Ran into cold-treated mitotic embryos results in complete cessation of chromatin-induced spindle formation (Hayward and Wakefield, 2014; Hayward et al., 2014). Therefore, it is possible that dHURP has evolved to regulate Aurora A in the absence of full-length TPX2. Alternatively, there may be an as-yet unknown link between Ran, Aurora A and other regulators such as Aurora Borealis (Bora) and Ajuba. In *Drosophila*, Bora activates Aurora A (Hutterer et al., 2006) and localization of Aurora A relies on Ajuba (Sabino et al., 2011). Bora is a good candidate for a Ran-regulated SAF, as it is retained in the nucleus during interphase (Hutterer et al., 2006), indicating the presence of a NLS. Both Bora and Ajuba have vertebrate homologs with roles in mitosis (Hirota et al., 2003; Hutterer et al., 2006), though Ran dependency has not been investigated. It may be that in vertebrates the TPX2 and Bora/Ajuba pathways function redundantly in Ran-dependent mitotic pathways, or it may be that, due to the limited functionality of dTPX2 in relation to human TPX2, the Bora/Ajuba pathway and/or dHURP have been co-opted to compensate for TPX2-mediated Aurora A activation.

Aurora B is an obligate part of the hetero-tetrameric Chromosomal Passenger Complex (CPC) together with INCENP, Borealin, and Survivin. Ran.GTP has been shown to directly bind Survivin in vertebrates (Xia et al., 2008), but surprisingly, dominant-negative Ran elicits no effect on CPC kinase activity (Kelly et al., 2007) suggesting Ran activity may be dispensable for CPC activity. Instead, the significance of the Ran-Survivin interaction appears deliver TPX2 to MTs (Xia et al., 2008). Whether Ran also interacts with Survivin in *Drosophila* is unknown; the truncation of dTPX2/Mei38 may abrogate binding. However, as the MT bundling activities of dTPX2/Mei38 appear to be conserved in *Drosophila*, a potential role for Ran/Survivin in loading dTPX2 on to MTs, at least during mitosis, may still be required.

The interactions between kinases and Ran function in mitosis are most likely broader than described above. For example, the Cyclin Dependent Kinase, CDK11, is involved in multiple aspects of cell cycle regulation, such as centrosome maturation and separation (Petretti et al., 2006) sister chromatid cohesion (Hu et al., 2007; Rakkaa et al., 2014) and cytokinesis (Wilker et al., 2007; Franck et al., 2011) and, in *Xenopus*, a long isoform of this kinase, p110, has been demonstrated to be regulated by Ran (Yokoyama et al., 2008). Although the *Drosophila* homolog of CDK11, Pitslre, has been identified as a regulator of Rho activity during cytokinesis (Gregory et al., 2007), any relationship between it and Ran has yet to be investigated.

## Spatio-temporal aspects of ran function

### Regulation of centrosome duplication and function

The Ran gradient model, in which RCC1 is sequestered exclusively at mitotic chromosomes, suggests that only low concentrations of Ran.GTP are present around the centrosomes and thus that Ran would play a minor part in centrosome function. However, it has been demonstrated that a pool of Ran remains associated with centrosomes throughout the cell cycle (Keryer et al., 2003) and proteins linked with Ran function at centrosomes are beginning to emerge. In human cell lines, Ran is maintained at the centrosome by association with the scaffolding protein AKAP450 (Keryer et al., 2003). It is difficult to tease apart the nature of the relationship between the two proteins during mitosis, as AKAP450 is also responsible for anchoring of several other molecules, including the kinases Casein Kinase 1 (CK1) and Cyclin E-Cdk2 to the centrosome (Nishimura et al., 2005). In addition, AKAP450 can anchor the MT nucleating complex γ-TuRC to the centrosome, promoting MT nucleation (Takahashi et al., 2002). Therefore, while loss of AKAP450 from the centrosome leads to a failure in centrosome duplication and defects in mitosis, it is unclear whether these effects are due to Ran, to CK1, to γ-TuRC or to other anchored proteins. The situation is even more complicated in *Drosophila*, where the closest identified homolog of AKAP450 is the Pericentrin-like protein, dPLP. dPLP is the only large fly protein with a PACT (Pericentrin/AKAP450 centrosomal targeting) domain in *Drosophila* (Martinez-Campos et al., 2004) and, as such, it is not entirely clear whether it is a homolog of pericentrin or AKAP450 or represents a functional composite of both. What is clear is that dPLP affects centrosome duplication (Dobbelaere et al., 2008) and has an important role in the structural maintenance of centrosomes, though this seems to be primarily in interphase (Müller et al., 2010; Lerit et al., 2015; Richens et al., 2015). Whether this role is due to direct effects of dPLP or whether, like AKAP450, it acts as an anchoring protein for other kinases, and whether Ran is in any way involved, all remains to be seen.

A second centrosomal protein that has a clear functional relationship to the Ran pathway is Nucleophosmin (NPM/B23). Originally described as a nucleolar phosphoprotein (Okuda et al., 2000; Tarapore et al., 2002), NPM contains both an NLS and an NES, the latter of which is recognized by the Exportin Crm1, which shuttles the protein to centrosomes (Wang et al., 2005). Crm1 is required to maintain NPM at centrosomes (Shinmura et al., 2005; Wang et al., 2005) and NPM dissociation from centrosomes following phosphorylation by CDK2/Cyclin E triggers centrosome duplication at the onset of mitosis in mammalian cells (Okuda et al., 2000; Tokuyama et al., 2001). NPM is also an activator of Aurora A kinase at the centrosome at G2/M, suggesting potential cross-talk between the Ran and Aurora A pathways. Although there is a Nucleophosmin homolog in *Drosophila*, as yet there are no studies on its centrosomal or mitotic functions.

### The nuclear envelope

Most metazoan model organisms undergo mitosis having completely disassembled their nuclear envelope and their nuclear pore complexes. However, in *Drosophila* and *C. elegans*, which only disassemble the nuclear envelope at the spindle pole regions (Fuchs et al., 1983; Stafstrom and Staehelin, 1984; Harel et al., 1989; Katsani et al., 2008) nuclear pore disassembly is gradual, and is only complete at metaphase (Stafstrom and Staehelin, 1984).

The core of the nuclear pore complex is the Nup107–160 complex, consisting of Nup 37, Nup 43, Nup 85, Nup 96, Nup 107, Nup 133, Nup 160, She 1, and Sec 13 (Belgareh et al., 2001; Vasu et al., 2001; Harel et al., 2003; Loïodice et al., 2004). During interphase, this complex acts as a scaffold for the rest of the nuclear pore (Szymborska et al., 2013). During mitosis, however, Nup107–160 relocalizes to chromatin and recruits the MT nucleating complex γ-TuRC to the kinetochore (Mishra et al., 2010); a process which is Ran-dependent in *Xenopus* embryo extracts (Franz et al., 2007). A further nuclear pore complex subunit, Mel28/ELYS, has been identified as the initial building block for the nuclear pore; in vertebrates it is required for recruitment of the Nup107–160 complex to the reassembling nuclear pore (Rasala et al., 2006; Franz et al., 2007), and for recruitment of the same complex to the chromatin during mitosis (Galy et al., 2006; Rasala et al., 2006). However, Mel28/ELYS can directly recruit γ-TuRC to MT nucleation sites on the spindle, and this process is both independent of the Nup107–160 complex and essential for Ran-dependent spindle assembly (Yokoyama et al., 2014). Since, in *Drosophila*, the nuclear pores disassemble only gradually, the role of Nup107–160 in kinetochore attachment to MTs is unclear. Moreover, no research has, as yet, been undertaken on the Mel28/ELYS homolog in *Drosophila*, CG14215. However, in *C. elegans*, which also utilizes semi-open mitosis, Mel28 likewise localizes to the chromatin during mitosis and recruits the Nup107–160 complex (Galy et al., 2006).

Nup358/RanBP2 is also another nuclear pore complex protein, which during interphase is involved in nuclear transport (Figure 2; Wu et al., 1995; Yokoyama et al., 1995; Walther et al., 2002). During mitosis it associates with MTs, especially the spindle poles and kinetochores, and RNAi knockdown in HeLa cells results in metaphase catastrophe (Hashizume et al., 2013), and MT-kinetochore attachment defects (Salina et al., 2003). Although there is only minimal information on mitotic functions of Nup358/RanBP2 in *Drosophila*, it has been identified as a MAP, as a mitotic centrosomal protein and as an Ndc80 interactor using mass spectrometry (Przewloka et al., 2007; Hughes et al., 2008; Müller et al., 2010).

Following chromosome segregation, nuclear envelopes reform in order to generate two daughter nuclei. In *Xenopus*, karyopherins play an essential role, recruiting nuclear envelope vesicles to chromatin (Zhang et al., 2002b). As with other Karyopherin functions, this process is dependent on Ran, and Ran immobilized on beads is able to drive nuclear envelope reformation in *Xenopus* embryo extracts (Zhang and Clarke, 2000; Zhang et al., 2002a). The presence of Ran itself, however, is not sufficient for nuclear reformation to occur; addition of non-hydrolysable GTP inhibits this process, suggesting the GTPase cycle is important (Hetzer et al., 2000). Similarly to spindle assembly, it is theorized that, at late anaphase/early telophase, Ran.GTP releases nuclear envelope components around chromatin (Hetzer, 2010). Although the nuclear envelope does not fully disassemble in *Drosophila*, Ran is clearly involved in the dynamics of nuclear assembly. Injection of a mutant form of Importin β with reduced Ran.GTP binding affinity into *Drosophila* embryos stops Lamin from accumulating at the nuclear envelope (Timinszky et al., 2002), while addition of the same mutant to *Drosophila* embryo extracts inhibits nuclear envelope formation (Tirian et al., 2003). Therefore, although additional evidence is needed, it can be reasonably assumed Ran plays a similar role in nuclear envelope reformation following mitosis for both *Drosophila* and vertebrate systems.

### The spindle matrix

The idea of the spindle matrix, a persistent nuclear-derived cellular structure in the vicinity of the spindle, was first conceived in 1984 (Pickett-Heaps et al., 1984). The spindle matrix is thought to provide a physical framework upon which MTs or SAFs attach (Tsai et al., 2006; Johansen and Johansen, 2007; Zheng, 2010). While it is somewhat poorly defined, the perduring spindle-like localization of certain proteins following MT depolymerization and the presences of forces within the spindle region following laser-ablation of MTs suggest such a structure exists and has a role in spindle assembly (Johansen and Johansen, 2009).

The current literature suggests two distinct, yet possibly related, spindle matrices. In vertebrates, at the onset of nuclear envelope breakdown, Lamin B assembles into a matrix-like network that can retain SAFs after MT depolymerization (Tsai et al., 2006; Zheng, 2010). RNAi knockdown of the *Drosophila* homolog Lamin Dm0 produces monopolar spindles with low MT density, suggesting it has some similar function to the Lamin B derived spindle matrix (Goshima et al., 2007). In addition, it has recently been demonstrated that the conserved MT-associated protein, BuGZ, undergoes liquid-liquid phase transitions *in vitro*, driven by low complexity hydrophobic residues. This aggregation promotes spindle matrix assembly, driving MT concentration and spindle formation (Jiang et al., 2015). Intriguingly, in *Drosophila*, four proteins—Skeletor, Chromator, Megator, and East—have each been shown to localize as matrix components, where disruption of any of these results in disruption of the matrix and spindle defects (Johansen and Johansen, 2009). All contain low complexity FG repeats, and have been hypothesized to generate a gel-like colloid (Johansen and Johansen, 2009). While a vertebrate homolog of Megator exists [the Translocated Promotor Region (Tpr) protein] (Lince-Faria et al., 2009), the others appear to be *Drosophila*-specific (Johansen and Johansen, 2009). Irrespective of their precise molecular composition, there is evidence that both vertebrate and *Drosophila* spindle matrices are Ran-dependent; in *Xenopus* embryo extracts, Ran is required for assembly of Lamin B into the spindle matrix (Tsai et al., 2006) while injection of dominant-negative Ran into *Drosophila* embryos results in disruption of Skeletor localization (Silverman-Gavrila and Wilde, 2006). Thus, it seems likely that Ran plays a direct role in spindle matrix formation.

### MT-dependent MT nucleation

The robustness of bipolar spindle assembly is enhanced by Augmin, a conserved hetero-octomeric protein complex which binds to pre-existing MTs within the spindle, recruiting the MT nucleating complex γ-TuRC to generate further new MTs (Goshima et al., 2007, 2008; Hughes et al., 2008; Lawo et al., 2009; Uehara et al., 2009). The ability of *Xenopus* Augmin to generate branched MTs has recently been shown to be greatly enhanced in the presence of constitutively active RanQ69L and/or the Ran-mediated SAF TPX2 (Petry et al., 2013), strongly suggesting that complex activity is Ran-mediated. Further, inhibition of Augmin activity through injection of interfering antibodies into *Drosophila* embryos completely abolishes chromatin-driven MT nucleation following cold treatment (Hayward et al., 2014). Although it is possible that at least some aspects of this chromatin-driven pathway are Ran-independent, injection of dominant negative Ran (T24N) into cold-treated *Drosophila* embryos phenocopies Augmin loss (Hayward and Wakefield, 2014). Furthermore, RNAi against *Drosophila* Augmin in S2 cells has been shown to attenuate kinetochore-MT attachment, a Ran-dependent process (Bucciarelli et al., 2009). Together, this evidence suggests that Ran and Augmin act in the same pathways of chromatin-mediated spindle assembly and kinetochore-MT attachment, although the details have yet to be determined.

### Cytokinesis

There is some evidence that Ran plays a role in furrow formation and cytokinesis. Although cytokinesis does not occur in *Drosophila* syncytial divisions, pseudocleavage furrows form prior to the onset of anaphase, separating the dividing chromosomes from neighboring spindles and preventing nuclear fusion (Sullivan et al., 1990). These pseudocleavage furrows resemble cytokinetic furrows, and share many of the same components (Mazumdar and Mazumdar, 2002); thus, the mechanisms that regulate them are likely to be similar. In one of the first demonstrations of a role for Ran in furrow formation, injection of dominant-negative Ran (T24N) or Importin α into syncytial embryos was found to significantly reduce pseudocleavage furrow generation, correlating with a loss of Anillin and Peanut recruitment—proteins which are important for furrow stability (Silverman-Gavrila et al., 2008). These events occur distantly to the chromosomes, in metaphase, in a region where Ran.GTP is believed to be at a low concentration. However, there is evidence for a localized population of Ran.GTP at the cortex in humans (Wee et al., 2011) as discussed in Section Non-motor SAFs, which could drive an additional cortical Ran gradient independently of chromosomes. These results are somewhat confounded by a recent study using a temperature sensitive RCC1 cell line, also in humans, which suggests that chromosomally-derived Ran.GTP in anaphase is actually responsible for reducing the amount of cortical Anillin during asymmetric cell divisions (Kiyomitsu and Cheeseman, 2013). Moreover, the ability of Importin β2 to bind Anillin has been shown not to affect mitosis or cytokinesis; rather it required to sequester the protein in the nucleus in interphase, preventing abnormal cellular architecture (Chen et al., 2015). Although this could reflect differences between distinct populations of Anillin regulated through distinct Importins (see Section The Core Ran Pathway in *Drosophila* Embryos), such an explanation does not explain the discrepancy between a cortically-derived Ran.GTP gradient which may act positively upon Anillin and a chromosomally-derived Ran.GTP gradient that negatively regulates Anillin localization.

There is also indirect evidence for a second target of Ran during cytokinesis—the kinesin, KIF14. KIF14 is known to interact with the MT bundling protein PRC1, localizing to the central spindle in anaphase, to co-ordinate cytokinesis (Gruneberg et al., 2006). The germline-specific paralog of KIF14 in *Xenopus*, Nuclear and Meiotic Actin Bundling Kinesin (NabKin), which is required for cytokinesis during the second meiotic cycle, has recently been shown to be negatively regulated by Importin β and activated by Ran.GTP, strongly suggesting it as a canonical, but anaphase specific, SAF (Samwer et al., 2013). Interestingly, KIF14 in *Drosophila* is encoded by Klp38B, which has been shown to localize to condensed chromosomes and regulate spindle formation (Molina et al., 1997; Ruden et al., 1997). However, as a precise role in post-metaphase events has not been described, whether Klp38B is a conserved Ran target involved in central spindle formation and/or cytokinesis remains only a possibility.

Finally, as eluded to in Ran-dependent SAFs, there is evidence, albeit limited and again contradictory, that CDK11, which has a clear role in cytokinesis (Wilker et al., 2007; Franck et al., 2011), is regulated through Ran. Given the role of the *Drosophila* homolog, Pitslre, as a regulator of Rho activity during cytokinesis (Gregory et al., 2007), it is tempting to speculate that more Ran-dependent proteins with roles in cytokinesis remain to be discovered.

## Final remarks

Originally conceptualized as a molecular and chemical “GPS” for SAFs (Kalab and Heald, 2008), through the restriction of RCC1 to chromosomes, it is now becoming apparent that the spatially and temporally controlled generation of Ran.GTP in mitosis regulates events from centrosome duplication to bipolar spindle maintenance to reformation of the nuclear envelope post-mitosis. Although this helps to explain the pleiotropic effects of interfering with Ran function in mitosis, the many targets of the pathway hinder a detailed understanding of the molecular basis of these phenotypes. As such, we still do not have a complete understanding of how this signal coordinates a cellular response. Indeed, several important questions remain unresolved. First, is a Ran.GTP gradient essential for robust spindle formation or maintenance? Clearly levels of the RanGEF RCC1 may be greatly reduced without affecting spindle assembly, suggesting that different systems may tolerate a reduction in Ran.GTP to a greater or lesser degree and further supporting the notion that the robustness of mitotic spindle formation is achieved through the presence of multiple functional “modules,” whose input is tuned in each individual cell type and organism (Duncan and Wakefield, 2011). However, without RCC1 null mutants in multiple species, it is difficult to be absolutely clear about the universality of this pathway. Second, do most or all SAFs retain functionality in the interphase nucleus? As SAFs are translocated to the nucleus during interphase, the high concentration of Ran.GTP in the nucleus should result in their release from Importin complexes. Therefore, unless additional regulatory events such as mitotic phosphorylation are required, these SAFs ought to be active in the nucleus outside of mitosis. In support of this, several SAFs have, indeed, been shown to function in the nucleus during interphase (Jasin, 2002; Jaiswal and Narayan, 2008; Jiang et al., 2009; Reichert et al., 2011; Ohata et al., 2013; O'shaughnessy and Hendrich, 2013; Aydin et al., 2014; Neumayer et al., 2014; Vidi et al., 2014; Ha et al., 2015). Moreover, removal of NLSs from SAFs can result in interphase MT abnormalities (e.g., Chen et al., 2015). Such sequestration of already-active proteins in the nucleus prior to mitosis may therefore provide a way for cells to generate MTs quickly as soon as NEB occurs—a notion supported by the observation that dHURP immediately and preferentially localizes to those centrosomally-nucleated MTs facing the nucleus within the nucleus upon mitotic entry (Hayward et al., 2014). Thirdly, the ability of different Importins to sequester different SAFs and localize differentially suggests a complex and co-ordinated regulation of Ran.GTP in mitosis. Moreover, as a final curve-ball, it appears that Ran may not be the only GTPase whose activity releases SAFs. Very recently, it has been shown that, in humans, the RCC1-like protein, TD60/RCC2, acts as a GEF for the GTPase RalA. Loss of TD60, or expression of dominant-negative Ral into cells, results in multiple spindle defects (Papini et al., 2015).

In summary, the list of GTPase-dependent SAFs and their specific roles in mitosis, continues to grow. There are, therefore, many future avenues to explore, in order to fully appreciate the coordinated response of the cell to the dynamic Importin-SAF-Ran relationship. *Drosophila*, with its many experimental advantages, may yet provide one of the best systems in which to drive forward our understanding.

## Author contributions

The article was researched and written by JC, edited by AB and JW and revised by all authors. Figures were produced by AB.

## Funding

JC was funded by the BBSRC grant BB/K017837/1 to JW.

### Conflict of interest statement

The authors declare that the research was conducted in the absence of any commercial or financial relationships that could be construed as a potential conflict of interest.
